# Managing Mortality: Key Factors Influencing Hemiarthroplasty Outcomes in Geriatric Patients with Proximal Femur Fractures

**DOI:** 10.3390/medicina61040568

**Published:** 2025-03-22

**Authors:** Ercan Bayar, Tolgahan Cengiz, Şafak Aydın Şimşek, Bedirhan Albayrak, İsmail Büyükceran, Yılmaz Tomak

**Affiliations:** 1 Department of Orthopedics and Traumatology, Tosya State Hospital, Kastamonu 37300, Türkiye; 2 Department of Orthopedics and Traumatology, İnebolu State Hospital, Kastamonu 37500, Türkiye; tolgahancengiz@hotmail.com; 3 Department of Orthopedics and Traumatology, Faculty of Medicine, Ondokuz Mayis University, Samsun 55200, Türkiye; drsafakaydin@gmail.com (Ş.A.Ş.); bedirhanalbayrak.5595@gmail.com (B.A.); ismail.buyukceran@omu.edu.tr (İ.B.); ytomak@omu.edu.tr (Y.T.)

**Keywords:** hemiarthroplasty, proximal femur fractures, mortality, geriatric patient, survival rate

## Abstract

*Background and Objectives*: Proximal femur fractures represent a significant health issue in the elderly, associated with high morbidity and mortality rates. This retrospective cohort study investigated factors influencing mortality (age, gender, infection, mobilization status, hospital stay length, surgical delay) in patients undergoing hemiarthroplasty for proximal femur fractures in a tertiary university hospital in Turkey. *Materials and Methods*: A retrospective analysis was conducted on 481 patients who underwent hemiarthroplasty at 19 Mayıs University hospital in Turkey between 2012 and 2022, with final follow-up data collected in 2022. Data collected included demographic information, the type of surgical approach, the duration of surgery, comorbidities, postoperative complications, and mobilization status at 1 and 6 months post-surgery. Statistical analyses were performed using the Mann–Whitney U test for age and surgery delay; the Kruskal–Wallis test for comparisons of mortality subgroups; the Chi-square test for categorical variables such as gender, fracture type, anesthesia type, and postoperative complications; and the Z test for post hoc analysis of categorical data. *Results*: The overall mortality rate at final follow-up was 56.1% over the entire study period (2012–2022), with a 6-month mortality rate of 21.8% and a 1-year mortality rate of 33.2%. Age and male gender were significant predictors of mortality. Infection rates were significantly associated with higher mortality within the first year. The study found no significant relationship between surgical approach, duration of surgery, or anesthesia type and mortality. However, increased length of hospital stay and delayed surgery correlated with higher mortality rates. Mobilization status significantly impacted survival, with immobilized patients demonstrating the highest mortality rates. *Conclusions*: Mortality following hemiarthroplasty for proximal femur fractures is influenced by various factors, including age, comorbidities, infection, and mobilization status. Implementing strategies for early surgery and mobilization while maintaining strict aseptic techniques could potentially reduce mortality rates in this high-risk population.

## 1. Introduction

Hip fractures in people over 55 have become more common as the population ages and life expectancy rises [1]. These fractures are frequently observed in elderly patients with underlying osteoporosis and are associated with high complication rates, leading to a deterioration in quality of life. In addition to the increasing number of hip fractures, the surviving population is likely to be older and have more comorbidities, which contribute to more extended hospital stays [2]. These fractures are among the most common osteoporotic fractures and are associated with substantial morbidity and mortality. In general, hip fractures are common fractures encountered in orthopedic clinics, but this frequency varies depending on national health systems and demographic characteristics. For example, age- and sex-standardized hip fracture incidence rates range from 95.1 per 100,000 population in Brazil to 315.9 per 100,000 population in Denmark [3]. The incidence of these fractures increases with age and is particularly high in individuals with osteoporosis, frailty, and multiple comorbidities. Early surgical intervention and effective postoperative management play a crucial role in improving survival rates and functional outcomes.

Hip fractures show varying etiologies with age. While they are associated with high-energy trauma in younger patients, in elderly patients, even lower-energy traumas, such as falls from standing height, can result in hip fractures. Patients with hip fractures often present with multiple comorbidities, with 60% of such patients having these conditions at the time of admission [4]. Although there are regional differences depending on access to healthcare and treatment protocols, a study conducted in the UK found that the one-year mortality rate following a hip fracture is approximately 28%, compared with 21% in an age-matched population without hip fractures [4].

In recent years, despite significant advancements in the treatment of hip fractures in elderly patients, there is still no standardized treatment approach due to differences in both patients and surgeons. In the past, patients with femoral neck fractures were either left untreated or managed with traction. Many elderly patients died due to prolonged immobilization before fracture healing could occur, while those who survived often faced multiple complications. Today, due to the complications associated with conservative treatment in the elderly population, surgical intervention has become the gold standard. Approximately 98% of hip fractures are treated surgically [5]. A small portion of these patients have an unacceptably high risk of mortality related to surgery and anesthesia, making them ineligible for surgical intervention [6]. The purpose of surgery can be defined as enabling the patient to mobilize early and pain-free, facilitating care, reducing complications associated with prolonged bed rest, and allowing the patient to return to pre-fracture function as early as possible.

Femoral neck fractures are classified on the basis of the anatomical localization into subcapital, transcervical, and basocervical types, while intertrochanteric fractures form a separate category. These fracture types differ in terms of stability, treatment approach, and associated mortality risks. Additionally, fracture classification can be based on the materials used during treatment, with Type 1 fractures managed solely with prosthetic components and Type 2 fractures requiring additional fixation materials, such as cables or grip plates, to enhance stability.

Since this study aimed to investigate factors associated with mortality in patients with proximal femur fractures treated with hemiarthroplasty, key variables such as demographics (age, gender), surgical factors (surgical approach, duration of surgery), comorbidities, postoperative complications (infection, dislocation, revision surgery), mobilization status, length of hospital stay, and infection rates were assessed in relation to mortality. In this study, mobilization status was categorized into three groups: unsupported mobilization (patients walking independently), supported mobilization (patients requiring assistance such as a walker or caregiver), and immobilization (patients unable to mobilize even with support). This classification was used to evaluate its impact on postoperative mortality.

Surgical treatment options include internal fixation and arthroplasty. Arthroplasty options include hemiarthroplasty and total hip arthroplasty. Hemiarthroplasty is preferred in elderly patients and those with comorbidities due to its shorter operation time, reduced blood loss, lower transfusion requirements, and decreased incidence of dislocation compared with total hip arthroplasty [7]. This study aimed to explicitly address the research question: “What are the key factors influencing mortality after hemiarthroplasty in geriatric patients with proximal femur fractures?” By systematically analyzing demographic, surgical, and postoperative factors, this study sought to provide a comprehensive understanding of mortality predictors in this high-risk patient group.

## 2. Materials and Method

**Figure 1:**
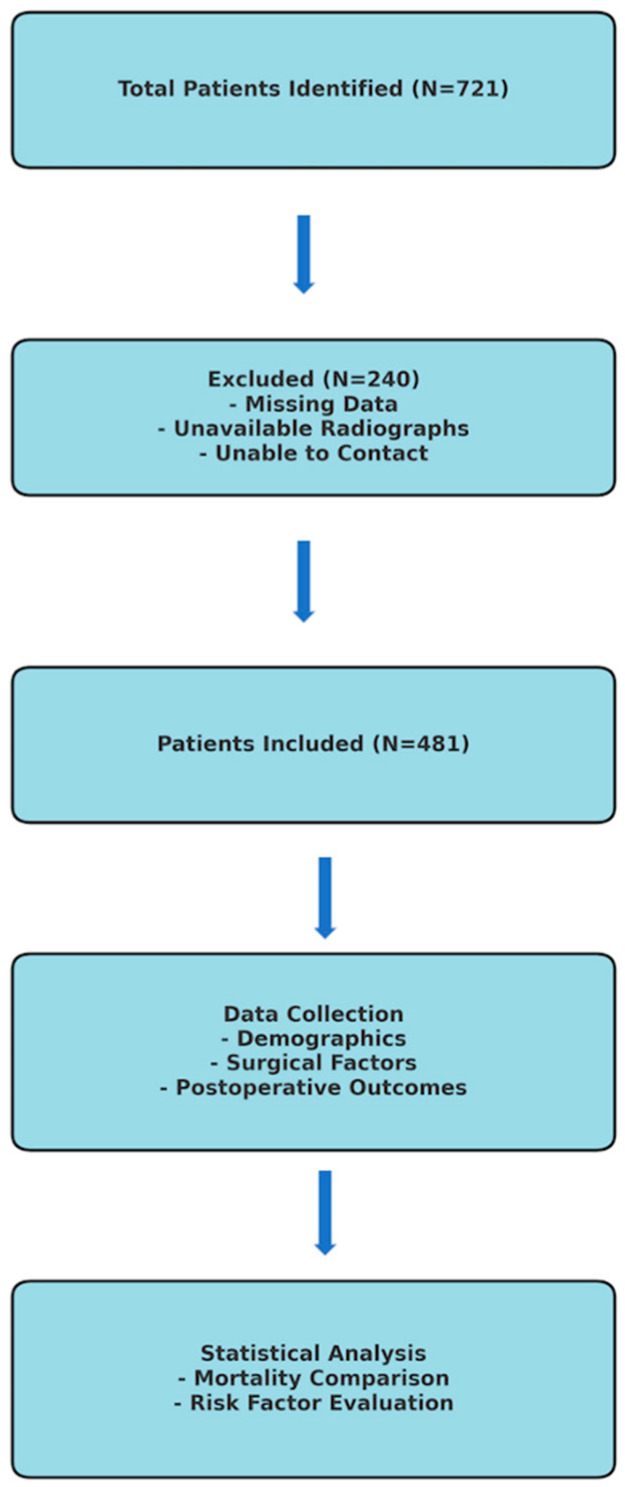
Flowchart during the planning phase of the study.

This study included patients who presented to the emergency department of a tertiary university hospital trauma center with a proximal femur fracture between 2012 and 2022 and were treated with hemiarthroplasty. Seven hundred and twenty-one patients were identified using the hemiarthroplasty surgery code from the hospital’s Mersis system. A total of 240 patients were excluded from the study due to the inability to access preoperative and postoperative radiographs, missing information in the hospital Mersis system, or inability to reach the patients or their relatives by phone. The study continued with the remaining 481 patients. A flowchart outlining the study design, patient selection criteria, and exclusion process is included in Figure 1.

Patients presenting to the emergency department underwent comprehensive evaluations, and informed consent was obtained from those recommended for hemiarthroplasty. After being admitted to the orthopedics and traumatology wards, patients received prophylactic treatment with low molecular weight heparin and anti-embolism stockings. Antibiotic therapy was administered preoperatively and postoperatively to reduce the risk of infection, and postoperative monitoring included fluid balance, vital signs, and routine hematocrit and biochemistry assessments. All patients received standard antibiotic prophylaxis before and after surgery. First-generation cephalosporin (2 g IV) was administered half an hour before surgery and continued for an average of 2 weeks until the stitches were removed (3 × 1 dose after surgery). During surgery, the surgical field was cleaned with povidone–iodine-based solutions to ensure a sterile surgical environment. In addition, sterile surgical drapes and glove changes were meticulously applied.

**Figure 2:**
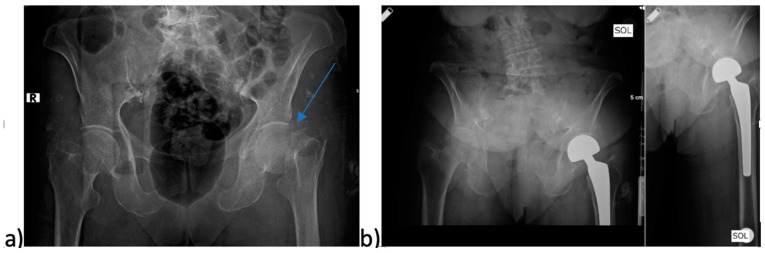
An 83-year-old female patient diagnosed with a Garden Type 4 fracture in the area indicated by the arrow in the left femoral neck, who underwent bipolar hemiarthroplasty and was mobilized on the second postoperative day. (**a**) Preoperative pelvis anteroposterior X-ray images; the fracture line is marked with the arrow. (**b**) Postoperative pelvis and left hip anteroposterior X-ray images.

Patients were mobilized early, and quadriceps exercises were encouraged as part of the rehabilitation process. Patients were guided in accordance with the standard physiotherapy protocol. This protocol included passive joint movements and sitting–walking trials in the first 24–48 h after surgery. The rehabilitation team carried out the physiotherapy process in the hospital environment and continued at home or in care centers after discharge. Mobilization levels of the patients were determined on the basis of walking capacity and assistive device use evaluated in the first and sixth months’ follow-ups after surgery. Patients were also educated on movement restrictions and assisted daily activities to prevent dislocation during recovery. In the postoperative follow-up, standard radiographs were utilized. These radiographs assessed the femoral stem angle, heterotopic ossification, acetabular wear, and femoral stem loosening. All cases were scheduled for physical examination and radiological evaluation on the 15th postoperative day, and at 1 month, 3 months, 6 months, 12 months, and annually after that. A case example included in the study is provided in Figure 2.

### 2.1. Inclusion and Exclusion Criteria

Patients who underwent hemiarthroplasty with lateral and posterolateral incisions due to proximal femur fractures between 2012 and 2022 at Ondokuz Mayıs University Faculty of Medicine, a tertiary care hospital in the Black Sea region of Turkey, were included in the study. However, those patients who did not have accessible data in the hospital information system, did not have pre-and postoperative anteroposterior pelvis and hip radiographs, or could not be contacted by phone, as well as those with pathological fractures, were excluded from the study.

### 2.2. Parameters Examined in the Study

Demographic characteristics (age, gender), surgical factors (type of surgical approach, duration of surgery, fracture type, time to surgery), and postoperative outcomes (intensive care follow-up, length of hospital stay, postoperative infection, postoperative dislocation and revision surgery history, type of anesthesia, and mobilization levels at the first and sixth months) were calculated and compared with mortality.

### 2.3. Statistical Analysis

Analyses were performed using the IBM SPSS 25 package program. Data were summarized as the mean ± standard deviation and median (min–max). The Kolmogorov–Smirnov test was used to check for the assumption of normality. The Mann-Whitney U test was used for pairwise comparisons where the normal distribution assumption was not met (e.g., comparing hospital stay duration in different mortality groups). The Kruskal–Wallis test was employed for comparisons of more than two groups (e.g., comparing age distributions among mortality subgroups). The Chi-square test was used to analyze categorical data (e.g., gender distribution in mortality groups). The Z test was performed for post hoc analysis of categorical variables (e.g., examining the significance of differences in mobilization levels and mortality). A *p*-value of <0.05 was considered statistically significant.

## 3. Results

**Figure 3:**
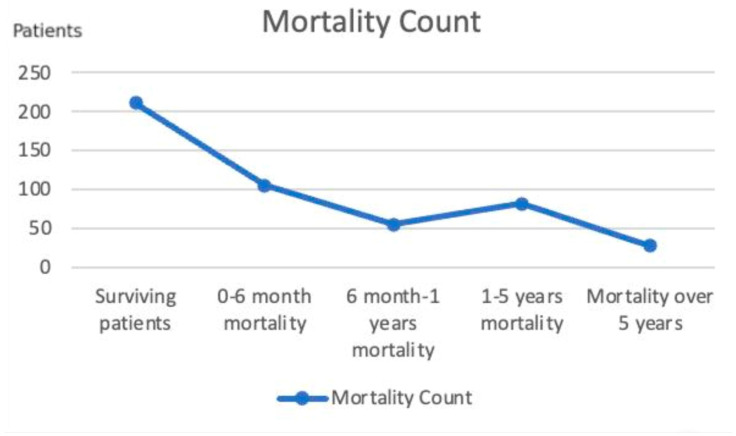
Mortality count of 481 patients by time periods in the Black Sea region of Turkey.

In our study, 160 (33.3%) of the 481 included patients were male, and 321 (66.7%) were female. The overall average age of these patients was 78.56 ± 8.82 years. The average age of male patients was 77.95 ± 7.76 years, while the average age of female patients was 78.87 ± 9.30 years. When examining the mortality rates in the study group, the overall mortality number was 270 (56.1%), while the number of surviving patients was 211 (43.9%). The deceased patients were classified into four categories. The mortality count in the 0–6-month period was 105 (21.8%), 55 (11.4%) in the 6-month–1-year period, 82 (17%) in the 1–5-year period, and 28 (5.8%) in the over 5-year period. According to this grouping, the highest mortality occurred in the 0–6-month period, followed by deaths in the 1–5-year period, the 6-month–1-year period, and over 5 years. Based on these values, the 6-month mortality rate was 21.8%, the 1-year mortality rate was 33.2%, and the 5-year mortality rate was 50.2% (Figure 3).

### 3.1. Demographic Factors and Mortality

Age with mortality was compared by calculating the mean and median values for each of the five mortality groups. The mean age of patients with 0–6-month mortality and those with 1–5-year mortality was found to be significantly higher than the other mortality groups (*p* < 0.001). Among these two groups, the mean and median ages of patients with 0–6-month mortality were higher than those with 1–5-year mortality (Table 1). In the comparison of mortality with gender, the survival rate of women was found to be significantly higher than that of men (*p* = 0.045). Men had a significantly higher 6-month to 1-year mortality rate compared with women (*p* = 0.045). No significant difference was found between men and women in the 0–6-month, 1–5-year, and over 5-year mortality groups (*p* > 0.05).

### 3.2. Surgical Factors and Mortality

Hemiarthroplasty was performed using the posterolateral approach in 350 patients (72.8%) and the lateral approach in 131 patients (27.2%). No significant association was found in the comparison between mortality and the surgical approach (*p* > 0.05). According to this result, the type of surgical approach does not affect mortality. In our study, dislocation rates in patients who underwent surgery via posterolateral and lateral approaches were compared, and no significant relationship was found between them (*p* = 0.769).

In our study, the mean duration of surgery for the 481 patients was 74.23 ± 27.42 min, with a median of 75 min (range: 20–180 min). No significant difference was found among the five mortality groups (*p* = 0.104) when comparing surgery duration with mortality rates. According to this result, the length of the surgery does not affect the mortality rate.

The patients’ average time to surgery after fracture was 3.05 ± 2.66 days. When comparing the times to surgery after the fracture among the different mortality groups, significant differences were found between the 0–6-month (*p* < 0.001), 1–5-year (*p* = 0.027), and over 5-year (*p* = 0.006) mortality groups. According to these results, the time to surgery for patients with 0–6-month mortality was significantly longer compared with the other two groups. Patients who waited a shorter period before surgery tended to survive at least the first year, while those who waited longer were more likely to die within the first 6 months.

The preoperative and postoperative direct radiographs of the 481 patients were examined, and the types of fractures were determined from the preoperative radiographs. Two different classifications were performed for the fracture types. First, on the basis of the anatomical localization, the fracture types were divided into four categories: Type 1, subcapital femoral neck fractures; Type 2, transcervical femoral neck fractures; Type 3, basoservical femoral neck fractures; Type 4, intertrochanteric femoral fractures (Table 2). In the second classification, patients who underwent surgery using only prosthetic materials were classified as Type 1, while those who used additional materials such as domino cables and grip plates for fixation alongside prosthetic materials were classified as Type 2 (Table 3). Significant differences were found when comparing mortality with two different classifications of fracture types. In comparing mortality with the classification of fracture types based on the materials used in treatment, it was observed that the 0–6-month mortality rate was significantly higher in Type 2 fractures (*p* < 0.001). This indicates that patients who required additional materials during surgery had significantly higher mortality rates within the first 6 months. When patients who used additional material during treatment were compared with the fracture type classification made according to anatomical localization, there was a significant relationship between intertrochanteric fractures and the use of additional material (*p* < 0.001). According to this relationship, there was a significantly higher use of additional material in intertrochanteric fractures. Additionally, when comparing the classification of fracture types based on anatomical fracture localization with mortality, a significant relationship was found between Type 4 intertrochanteric femur fractures and 0–6-month mortality (*p* = 0.003). Therefore, intertrochanteric femur fractures significantly increased mortality within the first 6 months.

### 3.3. Postoperatives Outcomes and Mortality

The postoperative dislocation rates of the patients were examined, and postoperative dislocation was detected in 15 (3.1%) of the 481 patients. No significant relationship was found when comparing the mortality rates between patients with and without dislocation (*p* = 0.353). According to this result, the mortality rates of patients who experienced postoperative dislocation did not increase compared with those without dislocation.

Patients who experienced deep and superficial infections during the postoperative period were recorded. Postoperative infection was detected in 30 (6.2%) of the 481 patients. When comparing the mortality rates of patients with and without infections, it was found that patients with infections had significantly higher mortality rates in the 0–6-month and 6-month–1-year periods than those without infections (*p* < 0.001). Conversely, the survival rate of patients without infections was significantly higher than that of patients with infections (*p* < 0.001). These findings suggest that the presence of postoperative infection significantly influences mortality. Of 481 patients, 32 (6.7%) underwent revision surgery, while 449 (93.3%) did not. Of these 32 patients, 15 (46.8%) underwent revision surgery due to infection, 11 (34.3%) due to dislocation, 3 (9.35%) due to periprosthetic fracture, and 3 (9.35%) due to aseptic loosening. In patients who underwent revision surgery, infection was the most common reason for revision. When comparing the mortality rates between those who had revision surgery and those who did not, the mortality rate in the 6-month–1-year period was significantly higher in patients who underwent revision surgery (*p* = 0.005). Similarly, in our study, the survival rates of patients who did not undergo revision surgery were found to be significantly higher than those who underwent revision surgery (*p* = 0.005). This result shows that the long-term survival of patients who did not undergo revision surgery was significantly higher.

The median hospital stay for 481 patients was calculated to be 7 (1–61) days. Patients with a hospital stay of 8 days or more were classified as above average, while those with a hospital stay of 7 days or less were classified as below average. According to this classification, 260 (54.1%) patients had a below-average hospital stay, while 221 (45.9%) had an above-average stay. When comparing the mortality rates of patients with above-average and below-average hospital stays, it was found that the mortality rate for patients with an above-average hospital stay during the first 0–6 months was significantly higher compared with those with a below-average stay (*p* = 0.018).

When examining the patients’ mobilization status at their 1-month and 6-month postoperative follow-ups, those who were mobilized with the aid of a walker or a caregiver were classified as the supported mobilization group. Patients who could mobilize independently were classified as the unsupported mobilization group, while those who could not mobilize even with assistance were classified as the immobilization group (Table 4). The mortality rates of patients who were mobilized with support, those who were independently mobilized, and those who were immobilized were examined. It was found that the mortality rate of immobilized patients in the first month concerning the 0–6-month period was significantly higher compared with both the supported and independent mobilization groups (*p* < 0.001). In the first month, the survival rate of patients who were able to mobilize independently was significantly higher compared with both the supported mobilization group and the immobilized patients (*p* < 0.001).

Furthermore, the survival rate of patients supported during mobilization was significantly higher than that of immobilized patients (*p* < 0.001). These results indicate that there is an inverse relationship between mobilization and mortality. When comparing mobilization and mortality at the sixth month postoperatively, it was found that the mortality rates of immobilized patients in the 6–12-month period were significantly higher than in those who were supported and independently mobilized (*p* < 0.001). Additionally, the mortality rate of patients who were able to mobilize with support in the sixth month was significantly higher compared with those who were able to mobilize independently (*p* < 0.001).

When examining the anesthesia methods applied to 481 patients, it was found that general anesthesia was administered to 196 patients (40.7%), while spinal anesthesia was administered to 285 patients (59.3%). Upon investigating the mortality rates of patients who received general anesthesia and those who received spinal anesthesia, no significant difference was detected between the two groups (*p* = 0.445). According to this result, the type of anesthesia administered does not appear to influence mortality. When examining the postoperative intensive care follow-up of the 481 patients, it was observed that 191 patients (39.7%) were monitored in the intensive care unit, while 290 patients (60.3%) were transferred to the ward without intensive care follow-up. In comparing mortality rates with respect to postoperative intensive care monitoring, it was found that the mortality rate for patients monitored in the intensive care unit during the first 0–6 months was significantly higher than for those who were not monitored (*p* < 0.001). According to this result, patients who were monitored in the intensive care unit after surgery have a higher risk of mortality within the first 6 months.

## 4. Discussion

Femoral neck fractures are primarily observed in the elderly population. In this demographic, they typically result from low-energy trauma, while those in younger individuals are more often associated with high-energy trauma. The presence of comorbidities and a decrease in physical capacity contribute to an increased risk of falls among older people. Additionally, osteoporosis can lead to complex and unstable fractures in the weakened proximal femur [8]. All of these variables should be evaluated when planning treatment.

Numerous studies have investigated mortality in patients who underwent hemiarthroplasty following hip fractures. In a survey conducted in the Netherlands on patients who underwent hemiarthroplasty due to hip fractures, the one-year mortality rate was 28%, while the five-year mortality rate was 54% [9]. In another study by Chang et al., the one-year mortality rate for patients who underwent hemiarthroplasty was 32% [10]. In a community-based study conducted in Norway, the one-year mortality rate was 21.3%, while the five-year mortality rate was 59% [11]. When examining mortality rates, the results of our study are consistent with the findings in the literature. Aslan et al. found that the mortality rate increased by 11.7 times in the third year compared with healthy individuals of the same age [12]. As seen in this study, femoral neck fractures significantly increase mortality in these older patients. In the current study, the mortalities of the patients were determined, with an overall mortality rate of 270 (56.1%), and the number of surviving patients was 211 (43.9%). The six-month mortality rate was found to be 21.8%, the one-year mortality rate was 33.2%, the five-year mortality rate was 50.2%, and the total mortality rate during the follow-up period was 56.1%.

### 4.1. Demographic Factors and Mortality

In our study, when comparing mortality and age, we found that the mortality rate in the first 6 months increased as age increased. In the comparison of mortality by gender, the overall mortality rate for males was found to be significantly higher than that for females. In the literature, there are numerous studies on patients who underwent hemiarthroplasty for hip fractures that report increasing mortality rates with advancing age and higher mortality rates in males compared with females, which aligns with the results of our study [12,13,14,15]. Factors such as hormonal differences, bone health, cardiovascular protection, and healthcare use may contribute to higher mortality rates in men. Future studies may further examine these biological and environmental factors to contribute to the development of gender-specific post-fracture management strategies.

### 4.2. Surgical Factors and Mortality

The literature includes studies that compare surgical approach with mortality and report no significant relationship among them [16]. We found no significant relationship between surgical approach and mortality. Our study did not detect a significant relationship between surgical approach and postoperative dislocation. When evaluated from this perspective, it was expected that there was no significant relationship between surgical approach and mortality.

A comparison of anesthesia types revealed no significant differences in their impact on mortality. This finding is consistent with numerous studies reported in the literature [10,12,17].

Smith et al. noted that patients with shorter surgery durations had lower one-year mortality rates [18]. Chang et al. found no significant relationship between one-year mortality and surgery duration [10]. Our study did not find a substantial relationship between surgery duration and mortality. We attribute this to the lack of a significant deviation from the averages in the surgery durations within our patient series. However, it is a fact that excessively long surgery times can diminish surgical success. We believe surgeries in this high-risk patient population should be completed within standard durations without compromising basic surgical principles.

Patients who waited longer for surgery had higher mortality rates in the first 6 months. While there are studies indicating that the duration of waiting for surgery does not affect mortality [12,19], studies emphasizing that increased waiting time for surgery is associated with higher mortality rates are predominant [10,15,17,20]. Patients with prolonged waiting times for surgery are noted to have comorbidities that require anticoagulant, antidiabetic, and cardiac medications or additional examinations. Rapid and close communication with the diagnostic units and relevant specialty physicians will help shorten waiting times for surgery and reduce mortality rates. Although there are different results in the literature regarding the time to surgery, it is recommended that surgery be performed as soon as possible, preferably within 24 h [21] and no later than 36–48 h [22], in elderly patients with hip fractures.

In our study, two classifications were used for fracture type. In the classification of fracture types according to anatomical localization, we found that patients with intertrochanteric fractures had significantly higher mortality rates within the first 6 months. Intertrochanteric fractures were more complex fractures compared with subcapital, transcervical, and basocervical femoral neck fractures. Intertrochanteric fractures are more unstable, are accompanied by more soft tissue trauma, and may require more blood transfusions. These fractures required more additional material use compared with femoral neck fracture types. It was expected that intertrochanteric fractures would increase mortality in the early period due to both the complexity of the fracture and the increase in the need for additional material use, and indirectly the increased risk of infection. In addition, according to the classification of fracture type according to the material used in treatment, we found that the mortality rates within the first 6 months were significantly higher in fracture types where additional material was used in treatment. In the series of 92 patients conducted by Aslan et al., no significant relationship was found in the mortality rates of patients with intertrochanteric and femoral neck fractures [12]. Kenzora et al. reported a one-year mortality rate of 13% for subcapital fractures and 15% for intertrochanteric fractures [20]. Holt et al. classified hip fractures as intracapsular and extracapsular femoral neck fractures, finding significantly higher mortality rates in extracapsular neck fractures [23]. We did not find any studies in the literature that classified types of fractures where additional materials are used alongside prosthetic materials in hip fractures. The use of additional materials indicates that the fracture is more unstable and also increases the postoperative infection rate, which in turn raises mortality rates. In addition, fractures that require additional material are generally more complex and unstable. Prolonged surgery time may also indirectly increase infection rates. Delay in postoperative mobilization in these patients may be among the factors contributing to mortality.

### 4.3. Postoperative Outcomes and Mortality

When comparing the rates of postoperative infection with mortality, we found that infection significantly increased both the mortality rates within 0–6 months and those within 6 months to 1 year. Pollmann et al. reported that infection increased the 3-month and 1-year mortality rates following surgery [24]. Lee et al. found that infection significantly increased mortality rates during the first 30 days and within the first year [25]. Partanen et al. also found that infection increased mortality in the first year following surgery [26]. As observed, infection is a significant risk factor for mortality. Postoperative infections may increase mortality by causing sepsis in patients and indirectly increasing the risk of organ failure. In addition, surgical revision may be required in patients who develop an infection. Additional surgeries may both create physiological stress and increase the risk of complications in patients by prolonging the hospital stay. At the same time, the delay in mobilization after surgery due to infection may be considered as another indirect mechanism of mortality. Therefore, utmost care must be taken to adhere to asepsis–antisepsis rules during the surgical process, as this should be one of the fundamental principles in treatment.

Blanco et al. compared the dislocation rates with mortality in patients undergoing hemiarthroplasty after hip fracture and found significantly higher 1-year mortality in patients with at least one history of dislocation [27]. In other studies comparing dislocation with mortality, no significant associations were found between dislocation and mortality [28]. Although there is no consensus in the literature on this issue, we consider it beneficial to remember that dislocation is a complication associated with hemiarthroplasty surgeries. When comparing the relationship between mortality and dislocation, no significant association was found between the mortality rates of patients with and without dislocation.

Sexson et al. reported that the mortality rate in patients with postoperative complications was three times higher than in those without complications [29]. When comparing mortality with revision surgery, we found that the mortality rates in the first year for patients who underwent revision were significantly higher than those who did not. In our study, the reasons for revision were identified as dislocation, infection, aseptic loosening, and periprosthetic fracture, with infection being the most commonly observed cause. All identified reasons for revision are complications associated with hemiarthroplastic surgery, and it should be remembered that these complications affect mortality. Maximum effort to avoid complications will help reduce mortality rates.

Aslan et al. reported no correlation between hospital stay duration and one-year mortality in patients who underwent hemiarthroplasty following femoral neck fractures [12]. In contrast, Simunovic et al. associated shorter hospital stays with lower mortality rates due to earlier mobilization and fewer complications [30]. Similarly, Rae et al. associated shorter hospital stays with earlier surgical intervention and reported a reduction in mortality rates [31]. The results of our study are consistent with the literature; when comparing hospital stay duration with mortality, it was observed that longer stays were associated with increased mortality rates within the first 6 months. There are many factors that affect the length of hospital stay. Postoperative complications, infection, and additional comorbidities are some of them. In such patients with prolonged hospital stays, delays in postoperative rehabilitation programs can be considered as another factor contributing to mortality. Therefore, the findings obtained in our study emphasize the importance of optimizing the postoperative process and managing complications in the early period. In order to shorten the length of hospital stay, early mobilization protocols, infection prevention strategies, and multidisciplinary care approaches are recommended to be implemented more effectively.

When analyzing the relationship between mobilization levels at 1 and 6 months postoperatively and mortality, it is evident that higher levels of mobilization are associated with significantly improved survival rates, whereas reduced mobilization correlates with markedly increased mortality rates. According to the results of our study, levels of mobilization have a significant impact on mortality. However, there are studies in the literature that have found no significant relationship between levels of mobilization and mortality [20]. There are more studies that have found that as the level of mobilization decreases, mortality increases [13,14,19]. Performing a stable hemiarthroplasty that allows for early mobilization post-surgery results in a reduction in mortality rates. In elderly patients, immobility leads to sarcopenia, reducing independent mobility. In the long term, the inability of these patients to continue their daily activities may increase the burden of care and shorten life expectancy. In particular, delayed mobilization after fracture may trigger depression, cognitive decline, and social isolation in geriatric patients, reducing quality of life and therefore survival. At the same time, the risk of thromboembolic complications increases in the event of prolonged bed rest, contributing to mortality. Considering these reasons, early mobilization is very important in patients undergoing hemiarthroplasty after hip fracture.

In our study, when comparing postoperative intensive care admissions with mortality rates, it was observed that patients monitored in intensive care had higher mortality rates within the first six months. Although Miniksar et al. did not find a significant difference in mortality rates between patients who remained in intensive care and those who did not during the postoperative period [32], there are numerous studies in the literature that support our findings [10,13,14]. It is natural for patients monitored in the intensive care unit postoperatively to have higher mortality rates, as they generally consist of those with more comorbidities and higher surgical risks. Our responsibility is to be more cautious and meticulous with these patients.

According to these findings, the following recommendations can be made for orthopedic surgeons and healthcare professionals.

- Early surgical intervention: Patients should undergo surgery within 48 h of admission to minimize complications and improve survival rates.
- Strict infection control protocols: Preoperative and postoperative infection prevention strategies, including appropriate antibiotic prophylaxis and sterile surgical environments, should be strictly followed.
- Optimized perioperative management: Preoperative stabilization of patient conditions can improve surgical outcomes.
- Postoperative early mobilization: Encouraging immediate postoperative mobilization through physical therapy can significantly reduce mortality rates.
- Shorter hospital stays: Efforts should be made to discharge patients as soon as they are medically stable to reduce the risk of hospital-acquired complications.

The greatest strength of this study lies in its large sample size and comprehensive evaluation of multiple factors influencing mortality following hemiarthroplasty for proximal femur fractures. By including a diverse patient population treated over a decade, the study provides robust data on demographics, surgical approaches, and postoperative outcomes, enabling a thorough analysis of key determinants of mortality. However, the study’s main limitation is its retrospective design, which may introduce selection and information biases due to incomplete or missing data, particularly from patients excluded due to inaccessible records or loss to follow-up. In addition, pre-fracture mobilization levels, nutritional status, additional comorbidities, postoperative blood replacement requirements, and biochemical parameters were not comprehensively evaluated. Additionally, the reliance on a single-center dataset may limit the generalizability of the findings to other settings or healthcare systems.

## 5. Conclusions

This study highlights the multifaceted factors influencing mortality following hemiarthroplasty for proximal femur fractures, emphasizing the critical importance of early surgical intervention, effective mobilization strategies, and rigorous infection prevention. By aligning with existing clinical guidelines and offering additional insights into modifiable risk factors, our findings provide actionable recommendations for improving perioperative management in geriatric patients. Our findings demonstrate that while age, comorbidities, and fracture type remain uncontrollable risk factors, timely surgery, shorter hospital stays, and early mobilization can significantly improve survival rates. These findings provide clear guidance for orthopedic surgeons and healthcare professionals on the best practices for reducing mortality after hemiarthroplasty. We believe that following the principles of taking patients to surgery as soon as possible, completing the surgery in a reasonable time with utmost attention to asepsis–antisepsis rules, mobilizing them as early as possible and ensuring their discharge, avoiding the use of additional materials and complications during surgery as much as possible, and limiting the choice of hemiarthroplasty to stable intertrochanteric fractures in fractures other than femoral neck fractures will reduce mortality rates. In addition to confirming well-established risk factors, our study adds a regional perspective by showing how mortality patterns in Türkiye compare with global findings. Healthcare system factors, rehabilitation availability, and cultural considerations in post-hospital care may influence outcomes and should be further explored. These insights underline the need for standardized, multidisciplinary approaches to optimize perioperative care and mitigate modifiable risks. Future studies should focus on validating these findings in a multicenter setting to refine treatment protocols further and improve long-term outcomes. By implementing targeted strategies and fostering collaboration among healthcare providers, it is possible to significantly reduce mortality and improve the quality of life in this vulnerable patient population.

## Figures and Tables

**Table 1:** Comparison of mortality with age (mortality group/years).

Mortality/Age	Age/Mean ± SD	Age/Median (Min–Max)
No mortality	76.96 ± 8.92	78 (37–95)
0–6-month mortality	81.3 ± 10.11	84 (48–101)
6-month–1-year mortality	76.58 ± 7.70	78 (54–91)
1–5-year mortality	80.78 ± 6.89	82 (56–96)
+5-year mortality	77.79 ± 6.18	78 (66–90)
Overall	78.56 ± 8.82	79 (37–101)

**Table 2:** Fracture types based on anatomical localization (number of patients/percentage).

	Type 1	Type 2	Type 3	Type 4	Overall
Number of Patients/percentage	38 (7.9%)	110 (22.9%)	73 (15.2%)	260 (54.1%)	481 (100%)

**Table 3:** Fracture types based on treatment materials used (number of patients/percentage).

	Type 1	Type 2	Overall
Number of Patients/percentage	364 (75.7%)	117 (24.3%)	481 (100%)

**Table 4:** Number of patients and percentage of mobilization levels at postoperative 1-month and 6-month follow-ups.

	Supported Mobilization	Unsupported Mobilization	Immobilization	Overall
First postoperative month	339 (70.5%)	26 (5.4%)	116 (24.1%)	481 (100%)
Sixth postoperative month	168 (34.9%)	224 (46.6%)	89 (18.5%)	481 (100%)

## Data Availability

The data presented in this study are available on request from the corresponding author.
